# Ultrasound-Guided Dextrose Hydrodissection for Mixed Sensory–Motor Wartenberg’s Syndrome Following a Healed Scaphoid Fracture: A Case Report

**DOI:** 10.3390/diagnostics16010156

**Published:** 2026-01-04

**Authors:** Yonghyun Yoon, King Hei Stanley Lam, Jeimylo C. de Castro, Jihyo Hwang, Jaeyoung Lee, Teinny Suryadi, Anwar Suhaimi, Chun-Wei Kang, Jaeik Choi, Seungbeom Kim

**Affiliations:** 1 Department of Orthopaedic Surgery, Gangnam Sacred Heart Hospital, Hallym University College of Medicine, 1 Singil-ro, Yeongdeungpo-gu, Seoul 07441, Republic of Korea; hwangjihyo36@gmail.com; 2 Incheon Terminal Orthopedic Surgery Clinic, Inha-ro 489beon-gil, Namdong-gu, Incheon 21574, Republic of Korea; 2wo02wo0@naver.com; 3 International Academy of Regenerative Medicine, Inha-ro 489beon-gil, Namdong-gu, Incheon 21574, Republic of Korea; jic121@hanmail.net (J.C.); stplayer@naver.com (S.K.); 4 Board of Clinical Research, the International Association of Musculoskeletal Medicine, Kowloon, Hong Kong; painfreedoc22@gmail.com (T.S.); anwar@ummc.edu.my (A.S.); 5 International Academy of Musculoskeletal Medicine, MSKUS, 1035 E. Vista Way #128, Vista, CA 92084, USA; 6 The Faculty of Medicine, The University of Hong Kong, Hong Kong; 7 The Faculty of Medicine, The Chinese University of Hong Kong, New Territory, Hong Kong; 8 SMARTMD Center for Non-Surgical Pain Interventions, Makati 1205, Philippines; jeidec@yahoo.com.ph; 9 Research Department, Adventist University of the Philippines (A.U.P.), Puting Kahoy, Silang 4118, Philippines; 10 Department of Physical Medicine and Rehabilitation, Hermina Podomoro Hospital, North Jakarta 14350, Indonesia; 11 Department of Physical Medicine and Rehabilitation, Medistra Hospital, South Jakarta 12950, Indonesia; 12 Physical Medicine and Rehabilitation, Synergy Clinic, West Jakarta 11510, Indonesia; 13 Department of Rehabilitation Medicine, University Malaya, Kuala Lumpar 50603, Malaysia; 14 Department of PM&R, Taipei Medical University Hospital, Taipei 110301, Taiwan; pmrken0805@gmail.com; 15 Dr. Choi’s Rehab. Med. Daelim Building 303, Kojandong, Ansansi, Kyengido, Seoul 15476, Republic of Korea; 16 Miso Pain Clinic, 1569, Bongyeong-ro, Yeongtong-gu, Suwon-si 16703, Republic of Korea

**Keywords:** Wartenberg’s syndrome, superficial branch of radial nerve, dynamic ultrasound, scaphoid fracture, dextrose hydrodissection, peripheral neuropathy, radial nerve entrapment

## Abstract

**Background and Clinical Significance**: Wartenberg’s syndrome (cheiralgia paresthetica) is classically described as a pure sensory neuropathy of the superficial branch of the radial nerve (SBRN). However, in rare circumstances, dynamic mechanical irritation around the radial styloid may produce an atypical clinical phenotype with concurrent motor impairment, broadening the clinical significance of recognizing motion-related compression mechanisms. **Case Presentation**: A 35-year-old woman presented with persistent dorsoradial wrist pain and numbness, accompanied by progressive weakness of thumb extension, five years after a conservatively treated nondisplaced scaphoid fracture. Neurological examination demonstrated sensory loss in the SBRN distribution and Medical Research Council (MRC) grade 3/5 strength of the extensor pollicis longus (EPL). Nerve conduction studies revealed a markedly prolonged EPL motor latency (4.5 ms; normal ≤ 2.5 ms) with preserved sensory conduction. High-resolution ultrasound showed focal enlargement of the SBRN (cross-sectional area 0.13 cm^2^) and, critically, dynamic snapping of the nerve over the radial styloid that reproduced the patient’s symptoms. The patient underwent ten weekly sessions of ultrasound-guided hydrodissection with 5% dextrose. After treatment, the pain Visual Analog Scale improved from 8/10 to 0/10 and EPL strength recovered to MRC 5/5. Follow-up nerve conduction studies demonstrated normalization of EPL motor latency (2.1 ms), and repeat ultrasound confirmed resolution of SBRN enlargement and snapping. **Conclusions**: This case expands the phenotype of Wartenberg’s syndrome to include mixed sensory–motor involvement associated with dynamic SBRN snapping at the radial styloid. Dynamic ultrasound was pivotal for identifying the motion-dependent mechanism, and ultrasound-guided 5% dextrose hydrodissection achieved complete sensory and motor recovery as a minimally invasive and effective treatment option.

## 1. Introduction

Wartenberg’s syndrome, or cheiralgia paresthetica, is an entrapment neuropathy of the superficial branch of the radial nerve (SBRN), first described by Robert Wartenberg in 1932 [1]. It typically presents with isolated sensory symptoms—pain, paresthesia, numbness, and burning sensations—along the dorsoradial hand and the first dorsal web space, occasionally extending proximally into the forearm [1,2]. The estimated incidence ranges from 0.03 to 1.16 per 1000 person-years in cohorts undergoing upper-limb nerve conduction studies, with a notable female predominance, potentially due to anatomical or hormonal factors [3].

Anatomically, the SBRN becomes subcutaneous approximately 9 cm proximal to the radial styloid, emerging between the brachioradialis and extensor carpi radialis longus tendons. It then courses beneath the brachioradialis tendon, arches over the anatomical snuffbox, and divides into its terminal digital sensory branches [4]. This pathway is fraught with anatomical variations; up to 30% of cadavers show accessory branches, and 5–60% exhibit connections to the superficial ulnar nerve [5,6]. These variations may create anatomical substrates that predispose the nerve to compression. Fascial septa in this region can form tight subcutaneous tunnels, making the SBRN vulnerable to external compression from casts, splints, tight jewelry, blunt trauma, or iatrogenic injury [7,8,9,10].

While the SBRN is primarily a sensory nerve, its intimate anatomical proximity to motor branches in the distal forearm—particularly those innervating the extensor pollicis longus (EPL), which courses nearby [11,12]—creates an anatomical basis for the theoretical possibility of concurrent motor involvement [8]. The EPL tendon passes through the third dorsal compartment, and its motor branch, derived from the posterior interosseous nerve, lies near the SBRN as they cross the distal radius. This anatomical confluence renders both structures susceptible to the same compressive forces in the snuffbox region [12]. However, such mixed sensory–motor presentations are exceptionally rare and not well-documented.

Classic etiologies of Wartenberg’s syndrome are well established and most commonly involve external compression of the superficial branch of the radial nerve (SBRN). Reported causes include constriction from tight wrist braces or casts after fractures or sprains, hematoma formation within the anatomical snuffbox, iatrogenic injury during venipuncture, and chronic pressure from prolonged leaning on the radial aspect of the hand [13]. Postsurgical SBRN neuropathy is also a recognized complication after wrist procedures such as volar plating or external fixation for distal radius fractures, wrist arthrodesis or arthroplasty, and wrist arthroscopy, where hardware irritation, portal placement, or excessive retraction may compromise the nerve [14,15,16,17]. By contrast, neuropathic sequelae following nonoperative management of carpal fractures—particularly scaphoid fractures—appear to be rare [18]. The literature contains only limited reports, including two case series describing sensory-only SBRN involvement attributed to casting-related compression [19,20]. To our knowledge, motor branch involvement has not been reported in the setting of conservatively treated scaphoid fractures.

Motor deficits in the radial distribution are more often explained by posterior interosseous nerve syndrome, radial tunnel syndrome, or EPL tendon rupture rather than primary SBRN pathology [21,22]. Accordingly, the existing reports following scaphoid fracture immobilization have been confined to sensory SBRN neuropathy, and those reports have not documented electrophysiologically confirmed involvement of an EPL motor branch in the context of a healed scaphoid fracture.

We report a case of mixed sensory–motor Wartenberg’s syndrome that developed insidiously after conservative management of a scaphoid fracture. The diagnosis was supported by complementary findings from dynamic high-resolution ultrasound and nerve conduction studies, demonstrating motion-dependent snapping of the superficial branch of the radial nerve. The patient was treated with a series of ultrasound-guided perineural hydrodissections using 5% dextrose in water, resulting in complete functional recovery. This case broadens the clinical spectrum of Wartenberg’s syndrome and highlights the value of advanced imaging for diagnosing and treating elusive neuropathies.

## 2. Case Presentation

### 2.1. Patient Information

A 35-year-old, right-handed female office worker presented with a five-year history of chronic left dorsoradial wrist pain and numbness. Her history was significant for a nondisplaced left scaphoid fracture sustained five years prior after a fall on an outstretched hand. The fracture had been treated conservatively with a thumb spica cast for six weeks, followed by a removable splint. Radiographic and sonographic union was confirmed at one-year follow-up. Plain radiographs showed no residual scaphoid deformity or carpal malalignment. She had no history of diabetes, thyroid disorders, or other systemic conditions that might predispose her to neuropathy. Written informed consent was obtained from the patient for the publication of this case report and any accompanying images.

### 2.2. Clinical Findings and Timeline

The patient’s symptom timeline is illustrative of the insidious nature of her neuropathy. Sensory symptoms, including burning pain and dysesthesia in the first dorsal web space, began approximately four weeks post-injury, coinciding with the period of cast immobilization. The sensory symptoms peaked in intensity around three months post-fracture. Notably, progressive weakness in thumb extension developed insidiously around eight months post-fracture, without any specific acute traumatic event. She reported difficulty with tasks such as writing, lifting a plate, and using her thumb for manipulation.

Upon presentation to our clinic five years post-injury, physical examination revealed the following:

- Sensory Testing: Sensory impairment to light touch (using Semmes–Weinstein monofilament 2.83 g) and diminished two-point discrimination (>10 mm) specifically in the territory of the SBRN (first dorsal web space and radial border of the hand).
- Motor Testing: Motor strength of the extensor pollicis longus (EPL) was graded MRC 3/5, indicating anti-gravity strength with inability to overcome resistance. Strength in wrist dorsiflexion (extensor carpi radialis longus/brevis) and finger abduction (intrinsics) was normal (5/5).
- Provocative Maneuvers: Tinel’s sign was markedly positive over the anatomical snuffbox, eliciting radiating paresthesias. Phalen’s test and Finkelstein’s test were negative, helping to rule out carpal tunnel syndrome and de Quervain’s tenosynovitis, respectively.
- Pain Assessment: A Visual Analog Scale (VAS) score for her dorsoradial wrist pain was 8/10 at rest, worsening with activity.

No systemic or rheumatologic symptoms were reported, and a laboratory workup, including complete blood count, erythrocyte sedimentation rate, and C-reactive protein, was unremarkable.

### 2.3. Diagnostic Assessment

A comprehensive diagnostic workup was pursued to elucidate the pathophysiology.

Nerve conduction studies (NCSs) were performed using a Nicolet™ Viking EDX electrodiagnostic system (Natus Neurology Incorporated, 3150 Pleasant View Road, Middleton, WI 53562, USA) running Viking software (version 22) (Natus Neurology Incorporated, Middleton, WI, USA), with skin temperature maintained at 32 ± 1 °C. Sensory conduction studies of the SBRN, performed antidromically with recording electrodes over the snuffbox and stimulation 12 cm proximally, were within normal limits bilaterally for both onset latency and amplitude [23]. This finding initially argued against a classic SBRN neuropathy. In stark contrast, motor NCS of the EPL branch revealed a markedly prolonged distal motor latency of 4.5 ms (normal ≤2.5 ms) with a well-preserved compound muscle action potential (CMAP) amplitude of 3.4 mV. This pattern was highly suggestive of a focal demyelinating lesion without significant axonal loss. Needle electromyography was deferred as the NCS and clinical picture sufficiently localized the problem.

High-Resolution Ultrasound: Ultrasound was conducted using an Alpinion XC90 Eliete (ALPINION MEDICAL SYSTEMS Co., Ltd., Seoul, Republic of Korea) with a 7–18 MHz linear hockey stick transducer, which provides excellent resolution for superficial nerves [24,25].

**Figure 1:**
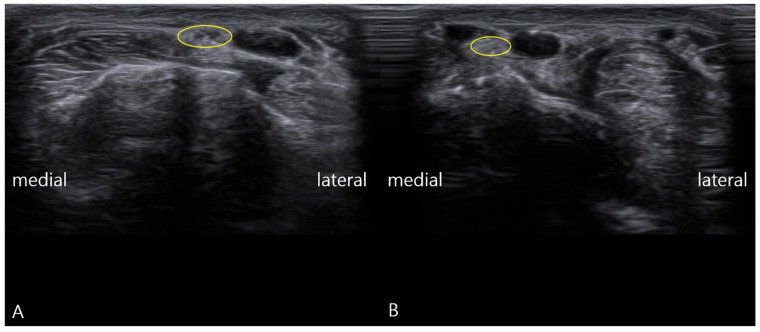
Transverse ultrasound images of the superficial branch of the radial nerve (SBRN): (**A**) pathologic left wrist showing fascicular thickening of the nerve (yellow circle); (**B**) healthy right wrist with normal fascicle size (yellow circle).

- Static Imaging: Transverse scans at the anatomical snuffbox revealed focal enlargement and loss of the normal fascicular echotexture of the SBRN on the symptomatic left side. The cross-sectional area (CSA) was measured at 0.13 cm^2^, significantly larger than the 0.08 cm^2^ on the asymptomatic right side (Figure 1). Power Doppler imaging showed mild hypervascularity around the left SBRN, suggesting ongoing inflammation.

- Dynamic Imaging: The patient was positioned supine with her wrist in neutral. The examiner then applied a shearing force to the radial styloid process while imaging the SBRN. This maneuver demonstrated palpable, visible snapping of the SBRN over the bony prominence of the radial styloid during ulnar deviation of the wrist (Supplementary Video S1). This dynamic finding directly reproduced the patient’s characteristic pain, providing a direct link between the anatomy and her symptoms [26,27]. In addition, a hand grip-and-release maneuver provided comparative dynamic assessment, showing normal findings on the contralateral side (Supplementary Video S2) and abnormal motion-dependent findings on the symptomatic side (Supplementary Video S3).

### 2.4. Therapeutic Intervention

**Figure 2:**
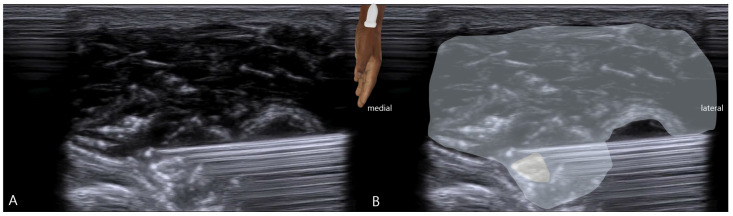
Ultrasound-guided hydrodissection of the superficial branch of the radial nerve (SBRN): (**A**) The patient’s hand is positioned in a neutral posture, as illustrated, and a 25-gauge needle is inserted from lateral to medial under real-time ultrasound guidance targeting the SBRN. The ultrasound transducer and scanning plane are illustrated in the center of the figure to demonstrate the procedural orientation. (**B**) Annotated image showing the superficial radial nerve highlighted in yellow and the injected 5% dextrose in water (D5W) solution depicted as a blue shaded region surrounding the nerve.

**Figure 3:**
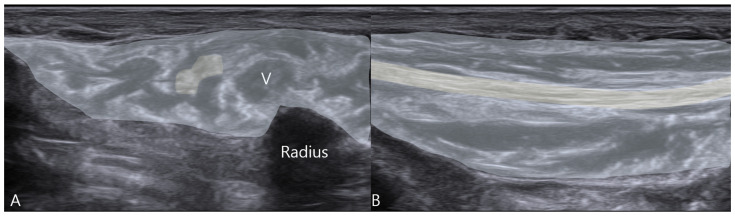
Post-hydrodissection ultrasound imaging of the superficial radial nerve: (**A**) short-axis view showing the nerve (yellow region) and the distribution of injected D5W fluid (blue shaded region), indicating nerve decompression; (**B**) long-axis view confirming expanded perineural space with hydrodissection fluid around the nerve.

Based on the diagnosis of a mixed sensory–motor Wartenberg’s syndrome with dynamic nerve snapping, a course of ultrasound-guided hydrodissection was proposed. The patient underwent ten weekly sessions. Under strict aseptic conditions, with the patient’s hand positioned in a neutral posture, using an in-plane ultrasound-guided technique, a 25-gauge 1.5-inch needle was advanced from lateral to medial, positioning the tip adjacent to the SBRN within the subcutaneous tissue plane (Figure 2). After negative aspiration, 20 mL of 5% dextrose in water (D5W) was injected slowly per session, with the objective of creating fluid expansion around the nerve, mechanically separating it from the surrounding adhesions and fascial constraints (Figure 3) [28,29,30]. The procedure was performed by the same experienced operator to ensure consistency.

### 2.5. Follow-Up and Outcomes

Clinical, electrophysiological, and sonographic improvements were monitored progressively and showed remarkable concordance:

- Pain and Sensory Outcomes: The patient reported progressive pain relief. Her VAS score decreased from a baseline of 8/10 to 4/10 after the third session, to 1/10 after the seventh session, and ultimately to 0/10 upon completion of the ten-session series. The burning dysesthesia in the first web space resolved completely.
- Motor Recovery: EPL strength improved steadily. It reached MRC grade 4/5 by the fifth session and normalized to full strength (5/5) by the eighth session.
- Electrophysiological Confirmation: Follow-up NCS performed six weeks after the final treatment session confirmed the functional recovery, showing normalization of the EPL motor latency to 2.1 ms, with a stable CMAP amplitude of 3.5 mV (Table 1).

### 2.6. EPL: Extensor Pollicis Longus; CMAP: Compound Muscle Action Potential

- Ultrasonographic Evidence: Post-treatment ultrasound revealed complete normalization of the SBRN’s appearance. The CSA decreased to 0.08 cm^2^, matching the asymptomatic side, and the fascicular echogenicity returned to normal. Most importantly, repeat dynamic assessment showed smooth, unimpeded gliding of the SBRN over the radial styloid without any snapping.

The patient successfully resumed light occupational duties by week six and returned to all pre-injury manual activities, including sports, by week eight without any recurrence of symptoms at a three-month follow-up. No adverse events, such as intraneural injection, infection, or post-procedural pain flare, were reported. Patient satisfaction was rated 10/10 on a standardized questionnaire.

## 3. Discussion

This case provides several profound insights that challenge conventional understanding and offer a refined approach to diagnosing and managing peripheral entrapment neuropathies.

### 3.1. Expanding the Clinical Phenotype of Wartenberg’s Syndrome

Our case compellingly demonstrates that Wartenberg’s syndrome is not invariably a pure sensory condition. The presence of significant EPL weakness, objectively confirmed by both physical exam (MRC 3/5) and NCS (prolonged motor latency), unequivocally indicates motor fiber involvement. This expands the clinical spectrum of the syndrome. The insidious onset of motor symptoms eight months after the initial injury suggests a chronic, progressive compression rather than an acute event. Clinicians should therefore include a careful motor examination of the EPL and other radial-innervated muscles in any patient presenting with dorsoradial wrist pain, particularly when there is a history of trauma or immobilization. Attributing thumb weakness solely to tendon pathology or posterior interosseous nerve palsy could lead to misdiagnosis and delayed treatment in such cases. This presentation is particularly noteworthy given the specific etiological context. While postsurgical SBRN neuropathy is documented [14,15,16,17], and transient sensory symptoms from cast compression have been reported [19,20], our case is unique in demonstrating a mixed sensory–motor deficit arising de novo years after a healed, nonoperatively managed fracture. This suggests that the pathophysiological mechanism extends beyond simple external compression or iatrogenic injury, implicating instead a persistent dynamic dysfunction arising from the healed fracture site itself.

### 3.2. Pathophysiological Insights: The Role of Dynamic Snapping

We propose a pathophysiological model in which subtle post-fracture functional changes may have contributed to motion-dependent mechanical conflict in the dorsoradial wrist. Plain radiographs demonstrated union without scaphoid deformity or carpal malalignment; therefore, the term “micro-instability” is used here to describe a dynamic phenomenon suggested by ultrasound during provocative maneuvers. Specifically, the hand grip-and-release test was normal on the contralateral side (Supplementary Video S2) but demonstrated abnormal motion-dependent findings on the symptomatic side (Supplementary Video S3). We hypothesize that these motion-dependent changes—potentially together with a prominent radial styloid and post-immobilization soft-tissue tethering—created a mechanical conflict, leading the SBRN to snap repetitively during wrist motion. The dynamic ultrasound finding was the cornerstone of this diagnosis, visually capturing the pathological mechanism [26,27]. The EPL motor branch, coursing within millimeters of the SBRN in this region, was likely subjected to the same shear and compressive forces during the snapping event, leading to focal demyelination and the observed conduction delay [8,12]. This mechanism mirrors other dynamic entrapment neuropathies, such as ulnar nerve subluxation at the elbow, where symptoms are provoked by motion despite normal static anatomy. This proposed mechanism of micro-instability-induced dynamic snapping differentiates our case from the classic etiologies of Wartenberg’s syndrome, which more commonly involve static compression from braces, casts, or surgical hardware [7,9,14,15,16,17]. It underscores that even a radiographically well-healed fracture can alter biomechanics sufficiently to create a new, symptomatic neural conflict over time, a consideration that has not been previously highlighted in the context of SBRN neuropathy following conservative fracture management.

### 3.3. The Critical Role of a Multimodal Diagnostic Approach

This case highlights the limitations of relying on any single diagnostic modality and underscores the power of an integrated approach.

- NCSs were essential for objectively confirming the functional impairment of the motor fibers (prolonged latency) and, surprisingly, ruling out significant sensory fiber dysfunction, which helped narrow the differential diagnosis. Notably, our findings are consistent with recent literature demonstrating that high-resolution ultrasound can reveal SBRN pathology even when electrodiagnostic studies are normal, as electrodiagnosis may miss focal nerve compression without significant axonal loss [25].
- Static ultrasound identified the structural correlate of the neuropathy (nerve enlargement and hypoechogenicity), but was insufficient to explain the specific symptom mechanism.
- Dynamic ultrasound provided the crucial kinematic link, directly visualizing the nerve snapping and reproducing the pain. It answered the “why” and “how” that the other tests could not [25]. In this context, dynamic ultrasound served as a vital problem-solving tool, uncovering the pathophysiological mechanism—nerve snapping—that was entirely invisible to conventional electrophysiology.

This synergistic combination of functional electrophysiology and dynamic anatomical imaging is paramount for diagnosing complex or atypical neuropathies, moving beyond a one-size-fits-all diagnostic pathway.

### 3.4. Hydrodissection and the Dual Mechanism of Dextrose

Ultrasound-guided hydrodissection with 5% dextrose proved to be a uniquely suited intervention for this condition. Its efficacy can be attributed to a dual mechanism of action:

1. Mechanical Neurolysis: The injected fluid creates a physical separation between the nerve and the surrounding constrictive tissues, lysing adhesions and effectively expanding the perineural space. This immediately alleviates mechanical compression and restores normal nerve gliding, as evidenced by the resolution of snapping on follow-up ultrasound.
2. Neuromodulatory Effects of Dextrose: The use of 5% dextrose, rather than saline or local anesthetics, adds a therapeutic pharmacological dimension. Prolotherapy theories and emerging evidence suggest that dextrose acts as a mild irritant that may stimulate healing and have neuromodulatory properties. It is postulated to stabilize neuronal membranes, reduce ectopic discharges, and modulate pain receptors like TRPV1, thereby reducing neurogenic inflammation and pain signaling [31,32,33,34]. Randomized trials in carpal tunnel syndrome have shown superior outcomes with dextrose hydrodissection compared to saline, supporting its bioactive role [29].

This combined mechanical and neuromodulatory action makes dextrose hydrodissection an ideal treatment for neuropathies involving both entrapment and neuroinflammation. Compared to traditional options—such as prolonged splinting, which may not address dynamic snapping, or surgical decompression, which carries risks of scar formation and iatrogenic injury—hydrodissection offers a precise, minimally invasive, and repeatable alternative with an excellent safety profile.

### 3.5. Limitations and Future Directions

The primary limitation of this report is its nature as a single case, which limits generalizability. The impressive outcomes, while robustly documented, could theoretically be influenced by the natural history or a strong placebo effect. Furthermore, the follow-up period of three months, while showing sustained recovery, is relatively short.

Future directions should include the following:

- Larger prospective cohort studies to determine the true prevalence of motor involvement in Wartenberg’s syndrome.
- Standardized protocols and diagnostic criteria for dynamic ultrasound assessment of peripheral nerves.
- Long-term studies to evaluate the durability of hydrodissection effects.
- Randomized controlled trials directly comparing dextrose hydrodissection with other established treatments, such as corticosteroid injection or surgical decompression, to establish evidence-based guidelines [29].

## 4. Conclusions

This case fundamentally expands the clinical spectrum of Wartenberg’s syndrome to include mixed sensory–motor presentations. It demonstrates that a healed scaphoid fracture can lead to dynamic nerve snapping as a novel pathophysiological mechanism. Clinicians should maintain a high index of suspicion for this entity in patients with persistent dorsoradial wrist pain and unexplained thumb weakness post-trauma. An integrated diagnostic approach, combining nerve conduction studies with dynamic high-resolution ultrasound, is essential for accurate diagnosis. Ultrasound-guided dextrose hydrodissection emerges as a highly effective, minimally invasive therapeutic strategy that addresses both the mechanical compression and the associated neurogenic inflammation, facilitating complete and sustained functional recovery. This case provides a compelling blueprint for the modern management of complex peripheral nerve entrapments.

## Figures and Tables

**Table 1:** Comparison of EPL motor nerve conduction parameters before and after hydrodissection.

Timepoint	EPL Distal Motor Latency (ms)	CMAP Amplitude (mV)
Baseline (Pre-treatment)	4.5	3.4
Post-treatment (6 weeks)	2.1	3.5

## Data Availability

All data supporting the findings of this study are available within the article and its Supplementary Materials.

## Supplementary Materials

The following supporting information can be downloaded at: https://www.mdpi.com/article/10.3390/diagnostics16010156/s1; Video S1: Dynamic ultrasound demonstrating snapping of the SBRN over the radial styloid during provocative maneuver. Video S2: Hand grip-and-release dynamic ultrasound test demonstrating normal findings on the contralateral side. Video S3: Hand grip-and-release dynamic ultrasound test demonstrating abnormal, motion-dependent findings on the symptomatic side.
